# Potential of Prebiotic Butyrogenic Fibers in Parkinson's Disease

**DOI:** 10.3389/fneur.2019.00663

**Published:** 2019-06-20

**Authors:** Thaisa M. Cantu-Jungles, Heather E. Rasmussen, Bruce R. Hamaker

**Affiliations:** ^1^Department of Food Science, Whistler Center for Carbohydrate Research, Purdue University, West Lafayette, IN, United States; ^2^Department of Nutrition and Health Sciences, University of Nebraska-Lincoln, Lincoln, NE, United States

**Keywords:** dietary fiber, Parkinson's disease, butyrate, gut microbiota, prebiotics

## Abstract

Parkinson's Disease (PD) is a neurodegenerative disorder characterized by loss of dopaminergic neurons in the substantia nigra. Recent evidence supports the involvement of the gastrointestinal tract in PD pathogenesis, including alterations in microbiota and intestinal permeability. Apart from being the preferred energy source for colonic epithelial cells, butyrate is involved in anti-inflammatory, enteroendocrine and epigenetic mechanisms that influence colonic and systemic health, including brain function. A few studies using oral administration of sodium butyrate indicate beneficial effects in PD animal models; however, prebiotic fibers that generate butyrate locally in the gut may be more effective. The design and selection of butyrogenic prebiotic fibers would allow preclinical studies to evaluate how gut-derived butyrate could affect PD pathophysiology. This review describes potential benefits of increasing gut butyrate production in PD through a prebiotic approach. Moreover, physico-chemical features of prebiotic fibers that target butyrogenic colonic bacteria are discussed.

## Introduction

Parkinson's disease (PD) is a relentlessly progressive neurodegenerative disease of aging, with a considerable burden of disability. It is believed that PD pathology is a consequence of both genetic susceptibility and toxic environmental factors, resulting in increasing neuronal oxidative stress (1). The pathological hallmark of PD is neuronal inclusions termed Lewy bodies (LB) or Lewy neurites (LN) whose main component is aggregated and phosphorylated α-synuclein and is responsible for neurological symptoms and signs of PD (2).

Gastrointestinal involvement in PD may be pathogenic or a consequence of the disease. More recently, researchers have provided evidence that supports a role for the gastrointestinal tract and the enteric nervous system (ENS) in the pathogenesis of PD (3, 4). α-Synuclein aggregates are present in Substance P containing neurons in the sigmoid colonic submucosal neurons in patients with PD (5). Microbiota differs between those with PD and healthy controls; for instance, those with PD have a lower abundance of *Clostridium* cluster XIVa and IV (6–10). Changes in caecum mucosal-associated and luminal microbiota, including a significant decrease in the relative abundance of the beneficial commensal bacteria genus *Bifidobacterium*, has been induced by a mouse model of PD (11). Recently, evidence for proinflammatory dysbiosis in PD patients has been shown, and researchers suggest that this dysbiosis could trigger inflammation-induced misfolding of α-Syn and development of PD pathology (6). Additionally, intestinal permeability was increased and beneficial metabolites of microbiota function, such as short chain fatty acids (SCFA), were lower in those with PD compared to healthy controls (5). As evidence for gastrointestinal tract involvement in PD exists, this suggests that therapeutic interventions may be warranted that positively impact the intestinal milieu by changing microbiota to produce less pro-inflammatory/injurious products and/or prevent gut leakiness.

## Prebiotic Fiber: Definition, Structure and Function

The term prebiotics was first introduced in 1995 by Gibson and Roberfroid as “a non-digestible food ingredient that beneficially affects the host by selectively stimulating the growth and/or activity of one or a limited number of bacteria in the colon, and thus improves host health” (12). Since then, the original definition has been revised several times and recently broadened to ‘a substrate that is selectively utilized by host microorganisms conferring a health benefit' (13).This should not be confused with probiotics, defined as “live microorganisms that confer a health benefit on the host when administered in adequate amounts” (14).

Although prebiotic definitions are general to all oligo- and polysaccharide prebiotic substrates, researchers up to 2010 have largely focused only on the use of fructans (fructooligosaccharides [FOS] and inulin), galactooligosaccharides (GOS) and, to a minor extent, lactulose, to promote beneficial shifts in the gut bacterial community (15). Prebiotic oligosaccharides were mainly used to promote increases in *Lactobacillus* and *Bifidobacterium* species (16). More recently, however, as the complexity and function of gut microbial ecosystems have been unveiled, new microbial groups or species of health interest have been identified, as well as ways to promote them (17–19). The challenge of achieving prebiotic effects favoring specific microbial groups requires the understanding of how prebiotic structure relates to substrate requirements of target bacteria and how they compete on substrates relative to other microbial groups (20).

The majority of prebiotic substrates fall into the dietary fiber classification—i.e., carbohydrate polymers not hydrolyzed by endogenous enzymes in the small intestine (21). Carbohydrates are the most abundant and heterogeneous class of molecules found in nature. In plants, non-cellulosic carbohydrate fibers include β-glucans, fructans, mannans, xylans, galactans, arabinans, arabinogalactans, pectins, and resistant starch. Also, carbohydrate fibers such as agars, sulfated carbohydrates, alginates, fucoidans, α,β-glucans and chitin may be found in other natural sources (22, 23). Apart from being a highly diverse class of molecules, complex variations at the fine chemical structure level (e.g., polymer size, linkage type, composition and arrangement of side chains, degree, and identity of ester-linked molecules) are possible within polymer class, resulting in dietary fibers with distinct solubilization degree, viscosity and tridimensional structure (20). For the complete hydrolysis and utilization of such complex molecules, a given gut bacteria should have within its genome the ability to produce recognition and binding proteins, transporters and carbohydrate-active enzymes (CAZymes) specific to a particular physicochemical structure (24). As such, the ability and efficiency in utilizing carbohydrates widely varies within gut individual bacteria or bacterial groups (24, 25). In addition, overlapping abilities in fiber degradation within bacterial species result in competitive pressures within the gut. For instance, Xu et al. (26) showed that strains of *B. cellulosilyticus* and *B. ovatus* both had the ability to grow on simple arabinoxylan structures. However, when the strains were cultivated together, *B. ovatus* outcompeted and dominated over *B. cellulosilyticus*. Thus, prebiotic fibers with specific physicochemical features can be selected to promote certain bacteria based on the ability of a bacteria or bacterial group to access and utilize them efficiently in the competitive environment of the colon (20).

## Metabolites From Colonic Dietary Fiber Fermentation in Parkinson's Disease

The colonic fermentation of dietary fiber by specialist microbes in the gut leads to the formation of a variety of gases and metabolites. SCFAs including acetate, propionate, and butyrate comprise 90–95% of all microbiota metabolites produced in the colon (27–29). SCFAs hold biological significance and may act both locally in the gut and systemically to promote health benefits at distinct body sites. In neurological disorders, SCFAs are potentially important for their role in anti-inflammatory processes (30–32), promotion of blood-tissue barrier integrity (33, 34), and neuromodulation (35, 36). Moreover, local effects such as triggering gut peristaltic reflexes (37) could be relevant, as constipation is an usual clinical finding in many neurological disorders, including PD (38, 39). Although there are no studies evaluating acetate and propionate singly in PD, butyrate has been studied and the majority of preclinical evidence suggests that it specifically could be beneficial in many aspects of PD (40–45).

### Butyrate

**Figure 1:**
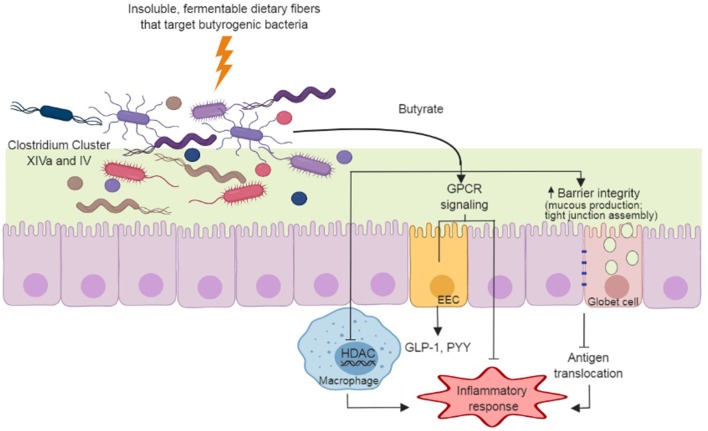
Dietary fiber approach to increase gut-produced butyrate and pathways that have potential benefits to Parkinson's Disease. Insoluble dietary fibers with specific chemical structures are fermented by butyrate producers in the gut (e.g., Clostridium Cluster XIVa and IV species). The butyrate produced during fermentation supports gut barrier function through the stimulation of mucus production and tight junction assemblies, and minimizing antigen translocation and inflammation. Butyrate also regulates inflammatory pathways through G protein-coupled receptors (GPCR) in enterocytes and through inhibition of histone deacetylase (HDAC) in macrophages. GPCR signaling in enteroendocrine cells (EEC) induce secretion of hormones (e.g., glucagon-like peptide-1 [GLP-1] and peptide YY[PYY]) that act in many organs, including the brain.

Butyrate is the preferred energy source for gut enterocytes, responsible for most of their energy metabolism (46). Butyrate also supports gut barrier function through the stimulation of tight junction assemblies and mucus production. As mentioned, hyperpermeability of the colonic epithelium occurs in PD (5); thus, the action of butyrate on the gut barrier may have clinical importance in PD. At the cell surface level, butyrate elicits a variety of physiological responses through G protein-coupled receptors (GPCR) in enterocytes (47). In particular, butyrate regulates inflammatory pathways that are important in maintaining gut homeostasis (48, 49) and stimulates the production of enteroendocrine hormones such as glucagon-like peptide 1 (GLP-1) and peptide YY (50, 51) (Figure 1). Both of these hormones reach circulation and exert their action through receptors spread at distinct body sites, including the brain. In a mouse model of PD, oral administration of sodium butyrate increased colonic GLP-1 levels as well as upregulated GLP-1 receptors (GLP-1R) in the brain and resulted in improved neurobehavioral impairment (52).

Butyrate also influences histone acetylation, a post-translational modification that influences the propensity of a gene to be transcribed or repressed (53). Butyrate acts as a histone deacetylase inhibitor (HDACi) (54), attenuating production and secretion of pro-inflammatory cytokines in response to lipopolysaccharide stimuli in macrophages, complementing analogous modulation of inflammatory process via GPCRs (55) (Figure 1). Butyrate-targeted histone deacetylase inhibition is also neuroprotective against dopamine cell death (44) and DNA damage (42) *in-vitro*. In a rotenone-induced drosophila model of PD, sodium butyrate was able to improve locomotor deficits and reduce early mortality (40). Similar results were observed in a 6-hydroxydopamine-induced rat model of PD, in which sodium butyrate attenuated motor impairment and increased dopamine levels (45). In addition, Zhou et al. (43) showed that in a cell culture and a murine model of PD, sodium butyrate was able to up-regulate gene expression of DJ-1, a protein known to protect dopamine neurons from oxidative stress and moderate protein aggregation.

All animal studies using PD models utilized oral administration of sodium butyrate, rather than an approach using butyrogenic prebiotics. It should be noted that sodium butyrate is delivered differently to the body compared to microbiota-produced butyrate from prebiotic fermentation. Sodium butyrate is absorbed mostly in upper segments of the gastrointestinal tract, it leads to significant increases in plasma concentrations of butyrate (56). While this could result in direct actions in the brain, upper gastrointestinal tract absorption prevents most of the butyrate supplemented to reach the large intestine, where it has functions that could be relevant in PD (e.g., gut barrier function, regulation of inflammatory pathways, enteroendocrine hormone release). Microbiota-derived butyrate, on the other hand, generally is considered to act locally in the gut, with the remaining portion absorbed by the liver with no significant amounts reaching bloodstream (57). Interestingly, some reports show increased blood levels of circulating butyrate in healthy subjects in response to dietary fiber interventions (58–60), indicating that a portion of butyrate may escape liver absorption and could have a direct action in the brain. Inflammatory conditions may also cause an increase of SCFAs in peripheral venous blood (61), and therefore, the extent of microbiota produced-butyrate that reaches bloodstream in PD patients is still a matter of investigation. Overall, the use of prebiotic dietary fibers to increase butyrate in the colon could promote both localized and systemic effects (Figure 1), which seems like a promising approach in the management of PD. However, preclinical studies are needed to evaluate how gut-derived butyrate affects PD pathophysiology.

Some controversy regarding the commonly accepted concept of anti-inflammatory and neuroprotective action of SCFAs was brought to light in a study using a mouse model of PD (62, 63). Sampson et al. (62) reported that the oral administration of a SCFA mixture, as well as a fecal transplant, to animals raised in a germ-free environment or antibiotic-treated, enhanced PD pathophysiology. It was not clear, however, if the SCFA mixture dosage utilized corresponds to levels that can be reached through gut-microbiota production. In this regard, oral administration of 100 mg/kg of sodium butyrate (NaB), but not 1,200 mg/kg, attenuated social deficits in an autism mouse model (64), indicating that distinct outcomes may take place by changing SCFA concentration. Another consideration is that orally delivered butyrate is mainly absorbed in the upper gastrointestinal tract and could have distinctly different outcomes from the colonic-produced butyrate.

## Butyrogenic Bacteria in the Large Intestine

A number of commensal gram-positive bacteria in the human gut possess the ability to produce butyrate. The majority of the butyrate producing bacteria belong to Clostridium Clusters IV and XIVa of the Firmicutes phylum. These clusters comprise highly oxygen-sensitive bacteria, which are estimated to significantly contribute to colonic butyrate production (65, 66). They also correspond to a numerically important portion of colonic bacteria. *Faecalibacterium prausnitzii* from Clostridium Cluster IV and *Eubacterium rectale* from Clostridium Cluster XIVa comprise up to 14 and 13%, respectively, of total fecal gut microbiota (67). Other major butyrogenic bacteria isolated from the human colon are *Roseburia* spp., *Eubacterium* spp., *Anaerostipes caccae, Butyrivibrio fibrisolvens, Coprococcus* spp. from Clostridium Cluster XIVa and *Subdoligranulum variabile* and *Anaerotruncus colihominis* from Clostridium Cluster IV (66). Many of commensal clostridial species preferentially colonize the mucus layer (e.g., *E. rectale, F. prausnitzii*, and *R. intestinalis*) which is in close proximity to gut epithelium. This strategic position favors butyrate interaction and uptake by intestinal cells, stimulating physiologic, metabolic and immunologic processes of health significance. Nonetheless, species such as *A. caccae* mostly inhabit the lumen of the colon where butyrate production helps to reduce luminal pH, preventing the growth pathogenic bacteria (68–70). Non-butyrogenic species also indirectly contribute to butyrate formation through production of other SCFA as a more acidic gut milieu favors the growth of butyrogenic species (71–73). Also, many butyrogenic bacteria utilize lactate and acetate from other bacteria to produce butyrate (66). The importance of such crossfeeding mechanisms to improve butyrate formation in the gut is still a matter of discussion as many butyrogenic bacteria occupy spatially distinct niches different than non-butyrogenic ones within the gut (70, 74, 75).

Depletion of butyrogenic bacteria from Clostridium Cluster IV and XIVa, especially those found nearly associated to the mucus layer is a common and potentially negative finding in the elderly (68). On top of that, PD patients show lower abundance of Lachnospiraceae family members (Clostridium Cluster XIVa) (6–8) and *Faecalibacterium* (Clostridium Cluster IV) (6, 8–10), as well as low production of all three SCFAs, including butyrate (9) compared to individuals of similar age.

## Prebiotic Dietary Fiber Targeting Butyrogenic Bacteria and Butyrate Production

Colonic bacteria produce butyrate mainly through dietary fiber fermentation, with proteolytic pathways contributing very little to overall butyrate production (65). Consumption of a meat-based diet for five consecutive days resulted in lower butyrate levels in fecal samples of healthy volunteers when compared to a plant-based diet. Butyrate reduction was accompanied by decrease in abundance of butyrogenic bacteria from Firmicutes, such as *Roseburia* and *E. rectale* (Clostridium Cluster XIVa). Another study with obese individuals showed that 4 weeks of a very low total carbohydrate intake (24 g/day), including low dietary fiber, resulted in a 4-fold decrease in *Roseburia* spp. and *E. rectale* accompanied by the same magnitude reduction in butyrate fecal content (76). These data suggest that these colonic bacteria are particularly dependent upon dietary fiber consumption.

Contrary to what is found in Bacteroidetes (known as carbohydrate generalists, as many species have overlapping nutrient utilization abilities), available data suggest that, in addition to crossfeeding, butyrogenic bacteria are more specialized to degrade unique fiber structures. For example, Sheridan et al. (77) showed that even bacteria from the same *Roseburia* genus (Clostridium Cluster XIVa) present variable abilities to grow in distinct substrates in single cultures, with little overlapping in fiber utilization capabilities within species.

As previously discussed, fiber physical features are also related to its fermentation profile. Most bacteria attached to particles recovered from human feces belong to Firmicutes (mean 76.8% against only 18.5% Bacteroidetes), with high abundance of species from Clostridium Cluster IV and XIVa (74). *In vitro* fecal fermentation of wheat bran also showed that Clostridium Cluster XIVa dominated amongst particle-associated bacteria (78). As primary colonizers of insoluble substrates, these bacteria would hold a competitive advantage to degrade insoluble fermentable substrates. In fact, in pure cultures of *R. intestinalis* and *Bacteroides xylanisolvens*, the former was shown to be strongly associated with insoluble xylan, while *B. xylanisolvens* was enriched in solubilized xylan fractions (79).

Corroborating these results, many insoluble substrates such as chitin-glucan and β-glucan, as well as some cereals rich in insoluble fractions, were shown to increase butyrate and/or colonic butyrogenic bacteria (Table 1). Chitin-glucan complexes were shown to specifically increase Clostridium Cluster XIVa, including *Roseburia* spp. in high-fat (HF) diet-induced obese mice and promoted desirable metabolic outcomes (80). In our research group, insoluble β-glucans from fungi specifically increased *Anaerostipes* spp. (Clostridium Cluster XIVa) from <0.5% of the total bacteria in the initial inoculum to approximately 24% after fermentation of such fiber *in vitro* (81). This was accompanied by butyrate increase from 12.5 to 24–26% after β-glucan fermentation (81). Whole grain barley (82) and wheat bran (83, 85) were shown to be fermented by members of Lachnospiraceae family (Clostridium Cluster XIVa) in human colonic microbiota. Lignocellulosic dietary fibers from feedstocks such as galactoglucomannan and arabinoglucuronoxylan were shown to increase *Faecalibacterium prausnitzii* (Clostridium Cluster IV) (84). In an indirect way, acetate producers, such as *Ruminococcus bromii* through utilization of resistant starch, can promote butyrate production through cross-feeding (87). These studies confirm that insoluble polymers with distinct chemical structures boost divergent butyrogenic bacteria in the colon. Finally, besides solubility degree and chemical structure, particle size may be an important fiber characteristic to consider in butyrogenic prebiotic fiber design and selection. Tuncil et al. (86) showed that *in vitro* fecal fermentation of larger wheat bran particle size fractions led to higher butyrate production, as well as increases in some members of the Lachnospiraceae family (Clostridium Cluster XIVa). In contrast, smaller particles were associated with higher propionate production.

**Table 1:** Examples of insoluble substrates capable of promoting butyrogenic colonic bacteria.

**Dietary fiber**	**Study design**	**Butyrogenic bacteria positively affected**	**Study**
Chitin-glucan complexes	Fecal analysis from diet-induced obese mice	Clostridium Cluster XIVa, including *Roseburia* spp.	Neyrinck et al. (80)
β-1,3/1,6-D-glucan	*In vitro* human fecal fermentation	*Anaerostipes* spp. and *Roseburia*	Cantu-Jungles et al. (81)
Whole grain barley	Fecal analysis from healthy human subjects	*Eubacterium rectale, Roseburia faecis* and *Roseburia intestinalis*	Martinez et al. (82)
Wheat bran	Fecal analysis from obese males	Members from Lachnospiraceae family	Salonen et al. (83)
Acetylated galactoglucomannan and highly acetylated arabinoglucuronoxylan (AGX)	*In vitro* human fecal fermentation	*Faecalibacterium prausnitzii*	La Rosa et al. (84)
Wheat bran	*In vitro* human fecal fermentation	Members from Lachnospiraceae family and uncultured butyrate producers	Duncan et al. (85)
Coarse wheat bran	In *vitro* human fecal fermentation	*Coprococcus eutactus, Roseburia* and other Lachnospiraceae family members	Tuncil et al. (86)

Overall, the few studies using dietary fiber treatment in PD patients have focused on intestinal constipation (39, 88) and its pharmacokinetic effects on drug absorption (88). Metabolites produced in the gut, and composition of gut microbiota in response to dietary fiber treatment, have not been assessed. Cross-sectional studies indicate that the microbial composition in PD patients present distinct composition from healthy controls (6–10). Although differences in microbial composition varies between PD and healthy controls across studies, all researchers report decreased abundance of butyrate producers, such as bacteria from Clostridium Cluster XIVa and/or IV (6–10). As butyrate is known to play important physiological roles both within the gastrointestinal tract and in diverse body sites, a dietary fiber approach targeting increases in colonic butyrogenic bacteria (Figure 1) could be beneficial to PD. Studies designed to evaluate dietary fiber effects on bacterial shifts and beneficial metabolite production, especially butyrate, as well as its relation to inflammation, gut permeability, and neurological outcomes in PD, should be conducted. Dietary fibers with specific chemical structures can be selected and/or designed to evaluate if a targeted colonic increase in butyrate and butyrate producers is beneficial to the management of PD outcomes beyond intestinal constipation.

## Conclusion

Promoting increases in gut-derived butyrate is a promising approach in PD that could have implications in the management of gut and systemic disturbances. Prebiotic fiber features such as solubility degree, and chemical and physical structures may be important in allowing butyrogenic bacteria to compete against Gram-negative carbohydrate-utilizing bacteria for a more targeted prebiotic approach. The use of specific butyrogenic prebiotic fiber structures in PD models would allow for future pre-clinical studies to understand the effect of gut-produced butyrate in PD.

## Author Contributions

TC-J and HR wrote the manuscript. HR and BH revised the manuscript.

### Conflict of Interest Statement

The authors declare that the research was conducted in the absence of any commercial or financial relationships that could be construed as a potential conflict of interest.
